# The Neuroglial Dialog Between Cannabinoids and Hemichannels

**DOI:** 10.3389/fnmol.2018.00079

**Published:** 2018-03-20

**Authors:** Valeria C. Labra, Cristian A. Santibáñez, Rosario Gajardo-Gómez, Esteban F. Díaz, Gonzalo I. Gómez, Juan A. Orellana

**Affiliations:** ^1^Departamento de Neurología, Escuela de Medicina, Facultad de Medicina, Pontificia Universidad Católica de Chile, Santiago, Chile; ^2^Centro de Investigación y Estudio del Consumo de Alcohol en Adolescentes, Santiago, Chile

**Keywords:** astrocytes, cannabis, neurons, connexins, pannexins, synaptic transmission, neuroinflammation

## Abstract

The formation of gap junctions was initially thought to be the central role of connexins, however, recent evidence had brought to light the high relevance of unopposed hemichannels as an independent mechanism for the selective release of biomolecules during physiological and pathological conditions. In the healthy brain, the physiological opening of astrocyte hemichannels modulates basal excitatory synaptic transmission. At the other end, the release of potentially neurotoxic compounds through astroglial hemichannels and pannexons has been insinuated as one of the functional alterations that negatively affect the progression of multiple brain diseases. Recent insights in this matter have suggested encannabinoids (eCBs) as molecules that could regulate the opening of these channels during diverse conditions. In this review, we discuss and hypothesize the possible interplay between the eCB system and the hemichannel/pannexon-mediated signaling in the inflamed brain and during event of synaptic plasticity. Most findings indicate that eCBs seem to counteract the activation of major neuroinflammatory pathways that lead to glia-mediated production of TNF-α and IL-1β, both well-known triggers of astroglial hemichannel opening. In contrast to the latter, in the normal brain, eCBs apparently elicit the Ca^2+^-activation of astrocyte hemichannels, which could have significant consequences on eCB-dependent synaptic plasticity.

## Introduction

Because neurons are the excitable cells responsible for the transmission of electrochemical impulses during major cognitive processes, they were considered for a long time the main functional units at the central nervous system (CNS; Navarrete and Araque, 2010). Nonetheless, accumulating evidence in the last two decades has showed that other cell types may also play an active and highly coordinated role in multiple brain functions (Iadecola, 2017). For example, microvascular endothelial cells are pivotal players in the neurovascular coupling, as they actively modulate both the local blood flow and transport across the blood-brain barrier (BBB), ensuring oxygen, glucose and metabolite supply on demand (Zhao et al., 2015). At the other end, along with being the major population of glial cells in the CNS, astrocytes also are the gatekeepers of the BBB and govern synaptic transmission, ionic and transmitter homeostasis, as well as antioxidant and neurovascular responses, among other functions (Allen and Eroglu, 2017). Although initially not considered an element of the chemical synapse, microglia are now recognized to have a broad spectrum of functions beyond their immune capabilities, ranging from neuronal pruning to synaptic plasticity (Li and Barres, 2017). Because they are the primary source of inflammatory mediators during infections, injuries and chronic neurodegenerative diseases, microglia finely control the inflammatory profile of astrocytes and decidedly impact the functions and fate of neurons (Kettenmann et al., 2011). Most of the abovementioned functions and features tightly depend on the complex interaction and intercellular synchronization among brain cells. In mammals, cell-to-cell communication is in part mediated by two unrelated families of plasma membrane proteins: connexins and pannexins (Wang et al., 2013a; Decrock et al., 2015).

**Figure 1:**
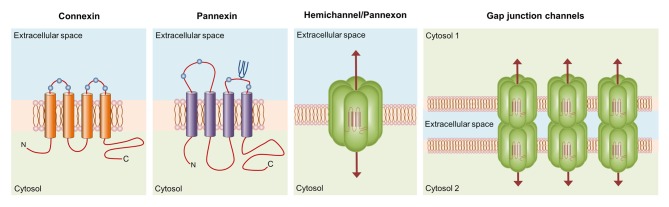
Basic structure of connexin and pannexin-based channels. Connexins and pannexins share a similar membrane topology with four α-helical transmembrane domains connected by two extracellular loops and one cytoplasmic loop; both the amino- and carboxy-termini are intracellular. The relative positions of the extracellular loop cysteines (green balls) and glycosylated asparagines (blue branches) are also shown. Hemichannels (also known as connexons) are formed by the oligomerization of six subunit connexins around a central pore. Pannexons are single membrane channels that are composed of six pannexin subunits. Recently, a band pattern more consistent with an octamer than a hexamer was observed in Panx2 by cross-linking studies and native gels of purified homomeric full-length and C-terminal truncation mutants (Ambrosi et al., 2010). Under resting conditions, hemichannels and pannexons remain preferentially closed, but they may be activated by diverse physiological and pathological conditions and offer a diffuse transmembrane route between the intra- and extracellular milieu. Hemichannels dock each other to form functional cell-to-cell channels termed gap junction channels (GJCs). GJCs aggregate in well-known anatomical structures called gap junctions to facilitate the intercellular cytoplasmic exchange of metabolites, second messengers and ions.

Connexins encompass a broad protein family of 21 members in humans (20, 37 and 17 in mice, zebrafish and frog, respectively), whose major structural features include the presence of four highly conserved transmembrane domains, two extracellular loops, intracellular N- and C-termini and a cytoplasmic loop linking the second and third transmembrane segments (Esseltine and Laird, 2016; Figure 1). These proteins form two types of functional plasma membrane channels: hemichannels and gap junction channels (GJCs). Each hemichannel is the assembly of six connexins around a central aqueous pore that allow the bidirectional flux of ions and molecules between the intracellular and intracellular milieu (Sáez et al., 2010; Figure 1). Additionally, hemichannels may diffuse freely to areas of cell-to-cell contact to align and dock with compatible hemichannels from a neighboring cell to complete the formation of GJCs. The arrangement and clustering of hundreds of GJCs at membrane appositions constitute the gap junction plaque, which provide a pathway of direct cytoplasmic communication between adjacent cells (Sáez et al., 2003). These channels are the building blocks of the electrically conductive link between two neighboring neurons (electrical synapse), as well as the functional syncytium of coupled astrocytes that underlie the homeostatic regulation of ions, transmitters and metabolites at the CNS (Orellana et al., 2009; Decrock et al., 2015). For example, the essential architecture of electrically coupled retinal circuits rely on the communication through GJCs, as they are broadly found in all cell types of the retina (Bloomfield and Volgyi, 2009). While the formation of gap junctions was initially thought to be the central role of connexins, the last decade had brought to light the high relevance of unopposed hemichannels as independent mechanisms for the selective release of relevant biomolecules (e.g., ATP, glutamate, PGE_2_) during physiological and pathological conditions (Giaume et al., 2013; Montero and Orellana, 2015; Gajardo-Gómez et al., 2017).

Pannexins, on the other hand, belong to a three-member family of chordate proteins homologous to the invertebrate gap junction proteins: the innexins (Bruzzone et al., 2003). Pannexins, unlike innexins and connexins, seem to have lost the capacity to directly couple contacting cells and thereby, the molecular and ionic interchange between the cytoplasmic and extracellular space is today widely recognized as their fundamental function. Although these proteins share some structural and topological features with connexins, their amino acid sequence and major post-translational modifications totally differ (Dahl and Keane, 2012). Single pannexin channels, also known as pannexons, allow the release of different paracrine molecules, including ATP, UTP, D-serine and glutamate (Bond and Naus, 2014). Pannexons formed by Pannexin 1 (Panx1), the most ubiquitous member of its family, are activated through direct protein-to-protein interactions with P2X_7_ receptors (P2X_7_R; Locovei et al., 2007), whereas the P2Y receptor (P2YR)-mediated rise in intracellular free Ca^2+^ concentration ([Ca^2+^]_i_) causes similar effects in these channels (Locovei et al., 2006). Consequently, the crosstalk between pannexons and purinergic receptors has been proposed to be crucial for the paracrine ATP release and amplification of cell-to-cell Ca^2+^ signaling (Wang et al., 2013a; Dahl, 2015). The latter is particularly true for astrocytes. They can communicate each other and with neurons by the spread of Ca^2+^ waves through two major interdependent mechanisms. One of them requires the physical interaction among contacting astrocytes and involves the diffusion through GJCs of cytoplasmic Ca^2+^-mobilizing second messengers such as IP_3_, cADP-ribose (cADPR) or Ca^2+^ itself (Scemes and Giaume, 2006; Orellana et al., 2012b). Alternatively, ATP produced by astrocytes may diffuse in a paracrine way to activate P2XRs/P2YRs, increasing the [Ca^2+^]_i_ and perpetuating the propagation of Ca^2+^ waves as new ATP is released into the extracellular space (Guthrie et al., 1999; Anderson et al., 2004). This mechanism of ATP-induced ATP release constitutes a critical pathway through which astrocytes exert their modulatory actions over neuronal activity (Chen et al., 2013; Shen et al., 2017).

Throughout the CNS there is a complex pattern of expression for connexins and pannexins, being highly dependent on developmental stage, brain region, cell type and brain homeostatic conditions (Söhl et al., 2000; Koulakoff et al., 2012; Gaete et al., 2014; Swayne and Bennett, 2016). Up to now, most studies indicate that hemichannels and pannexons play pivotal roles at the normal CNS, including the establishment of adhesive interactions, tolerance to ischemia, fear memory consolidation, glucose and redox sensing, chemoreception, BBB permeability, spontaneous electrical activity, synaptic transmission and neuronal migration (Orellana et al., 2016). Nevertheless, during acute and chronic brain damage, these channels behave abnormally, showing an increased activity and altering their permeability properties to different crucial biomolecules. The latter phenomenon has been hypothesized as a common hallmark reflecting the homeostatic unbalance observed in diverse neuropathological conditions such as Alzheimer’s disease (AD), epilepsy, ischemia and HIV-associated neurological disorders (Domercq et al., 2010; Orellana et al., 2011a; Decrock et al., 2015; Berman et al., 2016; Robel and Sontheimer, 2016; Giaume et al., 2017). While the mechanisms linked to hemichannel/pannexon dysfunction remain still obscure, some clues coming from recent studies argue in favor of the involvement of inflammatory and redox signaling in this process (Takeuchi et al., 2006; Retamal et al., 2007; Karpuk et al., 2011; Adamson and Leitinger, 2014; Retamal, 2014; Avendaño et al., 2015). The release of potentially neurotoxic compounds through hemichannels and pannexons has been insinuated as one of the functional alterations that negatively impact the progression of multiple brain diseases, turning the study and comprehension of this field in something of high relevance (Shestopalov and Slepak, 2014; Kim et al., 2016; Giaume et al., 2017; Malik and Eugenin, 2017; Rovegno and Sáez, 2018). Accordingly, many research groups are currently focused in developing new therapeutic and pharmacological tools to tackle the exacerbated activity of hemichannels and pannexons (Moore and O’Brien, 2015; Becker et al., 2016; Willebrords et al., 2017). Recent insights in this matter have suggested cannabinoids (CBs) as molecules that could counteract the opening of hemichannels and pannexons during neuroinflammatory conditions (Froger et al., 2009; Gajardo-Gómez et al., 2017). In this review, we first describe major features of the brain endocannabinoid (eCB) system and its critical role in synaptic transmission. Later, the possible neuroprotective actions of eCBs are discussed, in particular, the inhibition of the uncontrolled opening of glial cell hemichannels. Finally, we hypothesize how astroglial hemichannels may participate in eCB-mediated synaptic transmission in the healthy brain.

## The Endocannabinoid System at the CNS

Although cannabis (*Cannabis sativa*) has been widely used throughout history for medicinal, religious, recreational and social purposes, just in the past two decades research in the field has brought to light its multiple benefits (Corcos et al., 2005; Biswas et al., 2017; Mahvan et al., 2017; Yassin et al., 2017). In 1964, tetrahydrocannabinol (THC), the principal psychoactive constituent of cannabis, was isolated and purified, permitting the elucidation of its chemical structure (Gaoni and Mechoulam, 1964). Since then, many studies have been conducted to reveal the molecular mechanisms responsible for the psychoactive effects of cannabis-derived compounds, also known as phytocannabinoids (Zuardi, 2008). Late in the 1980s, as a result of decades of research on THC, Devane et al. (1992b) discovered the binding sites for the first CB receptor in the brain: CB_1_. Shortly after the cloning of the CB_1_ receptor (Matsuda et al., 1990), a second receptor called CB_2_ was isolated from human promyelocytic leukemia cells (Munro et al., 1993), whereas two eCBs agonists were identified: anandamide or N-arachidonoylethanolamine (AEA) and 2-arachidonylglycerol (2-AG; Devane et al., 1992a; Sugiura et al., 1995). Up to now, several molecules encompass the current thirteen-member list of eCBs (Pertwee, 2015), including O-arachidonoyl ethanolamine (Porter et al., 2002) and 2-arachidonyl glyceryl ether (Hanus et al., 2001); nevertheless, most studies have been focalized on AEA and 2-AG. Whether plant derived, synthetic or endogenous, CBs are lipid messengers that activate at least two G_i/o_ protein-coupled receptors; CB_1_ and CB_2_; and one ionotropic receptor: the transient receptor potential vanilloid 1 (TRPV1; Zygmunt et al., 1999). AEA predominantly acts as partial agonist of both CB_1_ and CB_2_ receptors and shows less relative intrinsic efficacy and affinity for CB_2_ than for CB_1_ receptors (Pertwee, 2010). On the other hand, 2-AG binds with the same affinity to both receptors and exhibits higher potency than AEA as CB_1_ and CB_2_ receptor agonist (Pertwee, 2010). AEA also binds and activates TRPV1s (Zygmunt et al., 1999), whereas 2-AG also binds GABA_A_ receptors (Sigel et al., 2011). In concert, eCBs, their receptors and regulatory synthetic (N-acylphosphatidylethanolamine phospholipase D [NAPE-PLD] and diacyglyerol lipase α [DAGLα]) and catabolic (fatty acid amide hydrolase [FAAH], monoacylglycerol lipase [MAGL] and others) enzymes constitute the triad usually referred as the eCB system (Battista et al., 2006). This systemic pathway plays key homeostatic functions in the CNS, as well as in multiple peripheral sites including skin, cardiovascular system, gastrointestinal tract, adipose tissue and liver (Pertwee, 2012).

At the CNS, AEA originates from the metabolism of NAPE by the activity of NAPE-PLD, being this process dependent on rising [Ca^2+^]_i_ upon depolarization and/or activation of ionotropic receptors (Luchicchi and Pistis, 2012). The biosynthesis of 2-AG from triacylglycerols requires the action of DAGL triggered by the activation of metabotropic receptors coupled to the PLCβ (Kreitzer and Regehr, 2002). Once postsynaptic neurons release eCBs into the synaptic cleft, they diffuse in a retrograde manner to activate presynaptic G_i/o_-coupled CB_1_ receptors, which result in the hyperpolarization of presynaptic terminals and as a consequence decreases neurotransmitter release (Ohno-Shosaku et al., 2001). The latter occurs at least by two major mechanisms of synaptic depression. At one end, short-term stimulation of CB_1_ receptors may lead to presynaptic inhibition of Ca^2+^ influx through N- and P/Q-type voltage-gated Ca^2+^ channels (Kreitzer and Regehr, 2002; Nimmrich and Gross, 2012), or activation of presynaptic A-type and inward rectifier K^+^ channels (Kreitzer and Regehr, 2002). At the other end, long-term activation of CB_1_ receptors may blunt the function of adenylyl cyclase (Childers and Deadwyler, 1996), resulting in the subsequent reduction of presynaptic cAMP levels and PKA activity (Chevaleyre et al., 2007). Synaptic signaling of eCBs ceases with their re-uptake and subsequent intracellular degradation. Inactivation of AEA takes place primarily by action of FAAH (Cravatt et al., 1996), whereas 2AG is degraded by the presynaptic enzyme MAGL and α/β-Hydrolase domain-containing 6 (Dinh et al., 2002; Marrs et al., 2010). Additionally, eCBs can act as non-retrograde messengers, where 2-AG may stimulate postsynaptic CB_1_ or CB_2_ receptors, whereas AEA may open postsynaptic TRPV1s (Castillo et al., 2012).

Most of the psychoactive effects of cannabis depend on CB_1_ receptors and their vast expression on neurons throughout the brain, including the basal ganglia, cerebellar cortex and hippocampus (Moldrich and Wenger, 2000). Despite of being characterized in some neurons (Onaivi, 2011; Stempel et al., 2016); CB_2_ receptors seem mostly found in glial cells at the nervous system (see below) and immune cell in the periphery, such as macrophages, neutrophils, lymphocytes, natural killer cells and monocytes (Galiègue et al., 1995; Buckley et al., 2000; Núñez et al., 2004). What remained as an open question for a long time is whether glial cells actually express CB receptors. Although early studies described the presence of CB_1_ and CB_2_ receptors in astrocytomas and primary astroglial cultures (Bouaboula et al., 1995; Sánchez et al., 1998; Molina-Holgado F. et al., 2002; Sheng et al., 2005), other studies did not find CB receptors in astrocytes (Sagan et al., 1999; Walter and Stella, 2003; Lou et al., 2012). In microglia, first reports showed an important expression of CB_2_ receptors in primary cultures and cell lines from different species (Carlisle et al., 2002; Facchinetti et al., 2003; Klegeris et al., 2003; Walter et al., 2003; Ramírez et al., 2005), however, CB_1_ receptors were rarely detected (Waksman et al., 1999). These contradictory results may reflect differences in the approaches used, including animal strains or species, antibody specificity and efficacy and cell conditions, as well as the fact that lower expression of CB receptors could make extremely difficult their identification by western blotting or immunohistochemistry (Onaivi et al., 2012; Metna-Laurent and Marsicano, 2015). In particular, several *in vitro* culture conditions influence glial function and inflammatory profile, such as type of isolation, culture medium, serum supplementation, medium changes, confluence, cell age, substrates and purity (Saura, 2007; Codeluppi et al., 2011; Stansley et al., 2012; Bohlen et al., 2017). Nowadays, most *in vitro* and *in vivo* evidence indicates that both astrocytes and microglia express CB_1_ and CB_2_ receptors in rodents (Gong et al., 2006; Navarrete and Araque, 2008; Palazuelos et al., 2009; Sagredo et al., 2009; Mecha et al., 2015; Navarro et al., 2018), dogs (Fernández-Trapero et al., 2017) and humans (Benito et al., 2005, 2007), thus playing critical roles in immunomodulatory responses and synaptic plasticity (Di Marzo et al., 2015; Oliveira da Cruz et al., 2016).

## Neuroprotective Actions of Cannabinoids Via the Inhibition of Hemichannels

Neuroinflammation is a pivotal determinant in the pathogenesis and progression of multiple acute and chronic neurodegenerative diseases. Microglial activation, reactive astrogliosis, production of inflammatory mediators (cytokines, chemokines, nitric oxide [NO], reactive oxygen and nitrogen species [ROS/RNS]), BBB breakdown and subsequent brain infiltration of circulating immune cells characterize this process (Becher et al., 2017). Both microglial activation and reactive astrogliosis constitute graded and multistage conserved glial reactions that counteract acute damage, restoring the homeostasis and limiting the brain parenchyma injury (Kettenmann et al., 2011; Pekny and Pekna, 2014). Nevertheless, during severe challenges and chronic brain damage, microglia and astrocytes may turn in uncontrolled source of inflammatory mediators rather than exhibiting a repair-oriented activity profile. While an efficient immune response is necessary to resolve brain threats, under the above circumstances, astrocytes and microglia may worsen disease progression by altering synaptic function, ion homeostasis, antioxidant defense and neuronal survival.

A growing body of data support the idea that eCBs are endowed with powerful immunoregulatory and anti-inflammatory properties, influencing both the CNS and peripheral tissues (Walter and Stella, 2003; Rom and Persidsky, 2013; Turcotte et al., 2015). eCBs and synthetic CB receptor agonists decrease the production of NO, ROS/RNS, free radicals and pro-inflammatory cytokines in activated glial cells, while facilitate the switching of dysfunctional microglia towards an anti-inflammatory phenotype (Waksman et al., 1999; Molina-Holgado E. et al., 2002; Molina-Holgado et al., 2003; Sheng et al., 2005; Mecha et al., 2015). Remarkably, brain levels of eCBs and glial CB receptors increase during neuroinflammation and neurodegenerative conditions, which may reflect self-neuroprotective and adaptive processes aimed at limiting the deleterious effects of inflammatory responses. In this line, CBs have been proposed as therapeutic tools to tackle several brain pathologies such as AD, multiple sclerosis (MS), Huntington’s disease (HD), traumatic brain injury (TBI), Parkinson’s disease (PD), among others (Kendall and Yudowski, 2016; Lu and Mackie, 2016). Supporting this notion, CB administration greatly mitigates the symptoms generated in animal models of MS (Lyman et al., 1989), HD (Palazuelos et al., 2009) and AD (Ramírez et al., 2005; Martín-Moreno et al., 2012), as well as a well-characterized model of chronic neuroinflammation produced by the infusion of lipopolysaccharide (LPS; Marchalant et al., 2007). Accumulating evidence suggests that neuroprotective actions of CBs depend on cellular and molecular events modulating the dysfunctional status of glial cells (Stella, 2004, 2010). At this point, one line of thought has argued that CBs may favor neuronal survival by inhibiting the uncontrolled activity of glial hemichannels and pannexons (Orellana et al., 2012c).

Inflammation has been established as a corner stone in the impaired function of hemichannels and pannexons not only in the CNS but also in peripheral organs (Kim et al., 2016; Crespo Yanguas et al., 2017). Just in the last 3 years a large list of inflammatory agents have been shown to exacerbate the opening of these channels in glial cells, such as cytokines (Abudara et al., 2015), growth factors (Garre et al., 2016), LPS (Avendaño et al., 2015), human immunodeficiency virus (Orellana et al., 2014) and ultrafine carbon black particles (Wei et al., 2014). Over the same period, similar findings have been found in multiple animal models of human disease, including amyotrophic lateral sclerosis (Almad et al., 2016), AD (Yi et al., 2016), hypercholesterolemia (Orellana et al., 2014), traumatic injury (Rovegno et al., 2015) and stress (Orellana et al., 2015). The release of cytokines from activated microglia represents a fundamental mechanism of Cx43 hemichannel activation in astrocytes *in vitro* (Retamal et al., 2007) and *ex vivo* (Abudara et al., 2015). In healthy conditions, the expression and activity of these channels is not high, but enough to ensure the release and influx of relevantly biological substances (Orellana et al., 2016). However, when astrocytes are cultured with primary microglia in presence of LPS, the opening of Cx43 hemichannels, determined from unitary current and dye uptake recordings, is prominently raised (Retamal et al., 2007). The latter response is emulated with conditioned media (CM) harvested from LPS-stimulated primary microglia, bringing up the idea that soluble factors released by microglia modulate astrocyte hemichannels (Retamal et al., 2007). ELISA, immunoneutralization and cytokine receptor blocking analysis reveal that IL-1β and TNF-α are indeed the soluble factors increasing Cx43 hemichannel opening in primary astrocytes (Retamal et al., 2007), which is consistent with previous studies showing that both cytokines reduce Cx43 expression and coupling between astrocytes (Même et al., 2006). Hence, IL-1β and TNF-α released by LPS-treated microglia regulate gap junctions and hemichannels in an opposing manner in cultured astrocytes (Retamal et al., 2007).

With this in mind, Froger et al. (2009) investigated whether endogenous (methanandamide [Meth], a non-hydrolyzable analog of AEA) and synthetic (WIN-55,212-2 [WIN] and CP 55,940 [CP]) CBs could modulate the release of IL-1β and TNF-α from LPS-treated microglia and their opposite effects on astroglial GJC and hemichannel function. They found that WIN, CP or Meth suppress the release of TNF-α and IL-1β by LPS-treated primary microglia (Froger et al., 2009), reinforcing previous reports describing that THC, as well as eCBs or synthetic CBs, blunt the expression and production of both cytokines in activated primary microglia (Puffenbarger et al., 2000; Facchinetti et al., 2003). More relevantly, these CBs prevented the astroglial uncoupling elicited by the following conditions: (i) LPS treatment in astrocyte-microglia primary mixed cultures; (ii) CM harvested from LPS-activated primary microglia; or (iii) mixture of IL-1β and TNF-α (Froger et al., 2009). Particularly, these preventive effects occurred in an additive way, indicating that WIN, CP and Meth likely act on different CB receptors. Coincident with this line of though, CB_1_ or CB_2_ receptor blockers abrogated with distinct pharmacological profiles the counteracting actions of WIN and Meth (Froger et al., 2009). The latter fit with the current notion of astrocytes expressing both CB_1_ and CB_2_ receptors (Benito et al., 2007; Navarrete and Araque, 2008; Sagredo et al., 2009; Navarro et al., 2018). In the same study, WIN and Meth were found to fully abolish the increase in Cx43 hemichannel activity in astrocytes triggered by the mixture of IL-1β and TNF-α (Froger et al., 2009). Contrary to the preventive effects observed in astroglial uncoupling, the CB-mediated inhibition of hemichannel opening was not additive and depended only on the activation of CB_1_ receptors (Froger et al., 2009).

As indicated above, exacerbated opening of glial hemichannels or pannexons may lead to the release of potentially neurotoxic compounds, resulting in neuronal alterations and subsequent cell death. In a follow-up study, Froger et al. (2010) tested the contribution of Cx43 hemichannel opening to NMDA-induced excitotoxicity in neuron/astrocyte co-cultures after treatment with TNF-α and IL-β. NMDA treatment significantly increased neurotoxicity in cytokine-treated co-cultures compared to the untreated ones, whereas this response did not occur in neurons co-cultured with Cx43 knock-out astrocytes or in the presence of Cx43 hemichannel blockers (Froger et al., 2010). At this point, the authors addressed the possibility that CB treatment could have a neuroprotective effect in NMDA and cytokine-induced excitotoxicity. Concordant with this notion, WIN completely prevented the increase in NMDA neurotoxicity produced by the mixture of TNF-α and IL-β (Froger et al., 2010). In the same line, recent studies by Gajardo-Gómez et al. (2017) reported that CBs ameliorate the amyloid-β peptide (Aβ)-induced neuronal death by preventing the dysfunctional opening of Cx43 hemichannels in astrocytes. Using time-lapse measurements of ethidium uptake, as well as patch clamp recordings, they showed that WIN, 2-AG and Meth, fully neutralize the Aβ-induced Cx43 hemichannel opening in cultured astrocytes and acute hippocampal slices. These responses were accompanied with a significant decline of the Aβ-induced production of IL-1β, TNF-α and NO in astrocytes (Gajardo-Gómez et al., 2017). Similar to that found by Froger et al. (2009), blockade of CB_1_ receptors with SR-141716A completely prevented the counteracting action of WIN, 2-AG and Meth over the Aβ-induced Cx43 hemichannel opening in cultured astrocytes. CBs also improved neuronal survival by mitigating the Aβ-induced excitotoxic release of glutamate and ATP linked to the opening of astroglial Cx43 hemichannels (Gajardo-Gómez et al., 2017). In discrepancy with primary cultures, in acute hippocampal slices the protective actions of WIN over hemichannel activity and neuronal death occurred via the activation of both CB_1_ and CB_2_ receptors, suggesting a cumulative action of CBs as they can act simultaneously in several cell types (Gajardo-Gómez et al., 2017).

**Figure 2:**
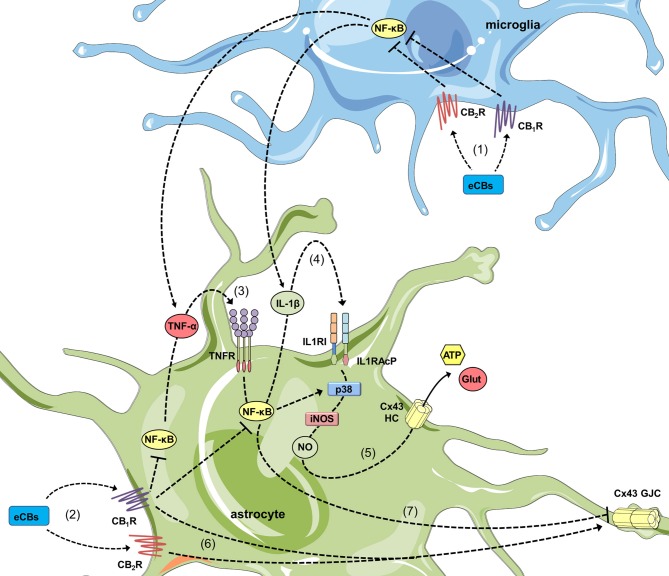
Cannabinoids (CBs) prevent the opposite regulation of astroglial connexin-based channels evoked by inflammatory conditions. In activated microglia, endocannabinoids (eCBs) acting on CB_1_Rs/CB_2_Rs counteract the NF-κβ-dependent release of TNF-α and IL-1β (1). In addition, in activated astrocytes, the stimulation of CB_1_Rs could also blunt the NF-κβ-mediated autocrine/paracrine release of TNF-α and IL-1β (2) along with the corresponding activation of TNFR1 (3) and IL1RI/IL1RAcP (4). The latter results in the inhibition of p38 MAP kinase and nitric oxide (NO) production, as well as the consequent reduction in excitotoxic release of gliotransmitters (e.g., glutamate and ATP) through astroglial Cx43 hemichannels (5). In parallel, activation of CB_1_Rs/CB_2_Rs may neutralize the reduction in gap junction communication (6) evoked by pro-inflammatory conditions (7).

How CBs impede the opening of hemichannels during pro-inflammatory conditions? A decade ago, Retamal et al. (2006) demonstrated that NO increases the opening of Cx43 hemichannels in ischemic astrocytes by inducing the S-nitrosylation of surface Cx43. Such key covalent modification also seems crucial for the cytokine-dependent opening of astroglial Cx43 hemichannels, as the inhibition of iNOS completely suppress this response in several pathological conditions (Retamal et al., 2007; Orellana et al., 2014; Avendaño et al., 2015; Gajardo-Gómez et al., 2017). In similar scenarios, blocking of p38 MAP kinase mitigates the activity of these channels, which is consistent with the fact that this pathway is a well-established cytokine target that induces iNOS expression and subsequent NO production in astrocytes (Wang et al., 2017; Figure 2). Altogether, these data denote that CB-mediated counteracting actions on hemichannel function may primarily proceeds by decreasing cytokine production and p38 MAP kinase activation, resulting in the subsequent suppression of NO production (Figure 2). In this context, the suppressive action of CBs on the NF-κβ pathway should be crucial, as it controls multiple aspects of neuroinflammation, including activation of glial cells and production of inflammatory mediators such as cytokines and NO (Shih et al., 2015; Figure 2). According with this notion, CBs blunt NF-κβ activation and reduce the production of NO and both IL-1β and TNF-α, as well as the activation of p38 MAP kinase in astrocytes (Curran et al., 2005; Sheng et al., 2009; Aguirre-Rueda et al., 2015), even in those stimulated with conditions that open hemichannels (e.g., Aβ; Aguirre-Rueda et al., 2015). Noteworthy, inhibition of NO production appears to rely on both CB_1_ and CB_2_ receptors (Sheng et al., 2005), while blockade of p38 MAP kinase activity is preferentially driven by CB_2_ receptors (Sheng et al., 2009). The latter may constitute a possible explication to the differential CB_1_/CB_2_ receptor pharmacology of synthetic and endogenous CBs in diverse astrocyte functions. A second but complementary mechanism of hemichannel regulation to that resulting from posttranslational modifications (e.g., S-nitrosylation) is the sorting of hemichannels to the cell surface. For instance, WIN fully abolish the Aβ-induced augment in surface levels of Cx43, signifying that modifications in surface expression of this protein may account for the preventive effects of CBs (Gajardo-Gómez et al., 2017). Future studies are required to elucidate the mechanisms associated to the CB-mediated regulation of surface Cx43.

## The Possible Crosstalk Between Astrocyte Hemichannels and Cannabinoid System During Synaptic Transmission and Plasticity

Without a doubt, the strategic alliance between neurons and astrocytes is crucial to understand the contemporary notion of synaptic transmission and plasticity (Araque et al., 2014). Pioneering studies by Araque et al. (1998a,b) served to later coin the term “tripartite synapse”, which describes how astrocytic processes together with pre- and postsynaptic terminals constitute a functional structure that sustain the activity of neural circuits (Eroglu and Barres, 2010). In this delicate physical and functional interaction, astrocytes sense and respond to neuronal activity by releasing bioactive molecules termed “gliotransmitters” through different pathways, including vesicles, P2X_7_R, volume-regulated anion channels (VRACs), bestrophin-1 Ca^2+^-activated chloride channels, pannexons, hemichannels and transporters (Gundersen et al., 2015). In addition to embracing the synaptic cleft, astrocytes are endowed with specialized terminal processes so called “endfeet”, which ensheath capillaries, intracerebral arterioles and venules; covering almost completely the abluminal vascular surface (Simard et al., 2003). This complex crosstalk with neurons and vascular cells provides astrocytes with an incomparable architectural advantage to facilitate local and long-distance release of gliotransmitters, thereby modulating synaptic transmission and plasticity with potentially significant consequences for memory and behavior (Dallerac and Rouach, 2016).

As already mentioned, retrograde eCB signaling may mediate short-term (Kreitzer and Regehr, 2001; Wilson and Nicoll, 2001) and long-term (Gerdeman et al., 2002; Marsicano et al., 2002) mechanisms of synaptic depression at both excitatory and inhibitory synapses (Navarrete et al., 2012). Surprisingly, although CB_1_ receptors are usually coupled to G_i/o_ proteins (Piomelli, 2003), the CB_1_-dependent [Ca^2+^]_i_ rise in astrocytes depends on the phospholipase C (PLC)-mediated IP_3_ production, indicating the involvement of G_q_ proteins rather than a “classical” inhibition of adenylate cyclase (Navarrete et al., 2012). Quite remarkably, eCB release from neurons requires astroglial Ca^2+^ elevations to stimulate glutamate release from astrocytes, which in turn, increases the frequency of postsynaptic NMDA receptor (NMDAR)-mediated slow inward currents (SIC) in proximal pyramidal neurons (Navarrete et al., 2012). A study from the same group described that [Ca^2+^]_i_ rise elicited by astroglial CB_1_ receptors lead to heterosynaptic short-term facilitation of synaptic transmission, likely through glutamate released from astrocytes and subsequent activation of presynaptic metabotropic glutamate receptors type-1 (mGluR1; Navarrete and Araque, 2010). These studies reinforce the idea that while eCBs trigger transient synaptic depression at local synapses via presynaptic CB_1_ receptors, they also induce transient synaptic potentiation at distant synapses through activation of astrocytic CB_1_ receptors.

The involvement of astroglial CB_1_ receptors in synaptic transmission also include processes of long-term plasticity. Indeed, stimulation of astroglial CB_1_ receptors increases [Ca^2+^]_i_ and causes glutamate release from astrocytes, which by activating presynaptic NMDARs trigger spike timing dependent long-term depression (t-LTD; Min and Nevian, 2012). Equivalent mechanisms of t-LTD have been found in the hippocampus, yet in these cases, either D-serine or glutamate seem the gliotransmitters involved in presynaptic or postsynaptic activation of NMDARs, respectively (Han et al., 2012; Andrade-Talavera et al., 2016). Noticeably, astroglial CB_1_ receptor-mediated t-LTD is critical for inducing the impairment of working memory *in vivo*, with the latter response being dependent on activation of NR2B-containing NMDARs and endocytosis of AMPARs (Han et al., 2012). Examples of astroglial participation in CB1 receptor-mediated long-term plasticity also include synaptic facilitation. Just a while ago, it was described that eCB-mediated activation of astroglial CB_1_ receptors elevates [Ca^2+^]_i_ and induces glutamate release that acting on presynaptic mGluR1s evokes long-term potentiation (LTP) when it coincides with the postsynaptic release of NO (Gómez-Gonzalo et al., 2015). Similar events of LTP facilitation have been described in the mouse neocortex with the ATP as major gliotransmitter implicated (Rasooli-Nejad et al., 2014). These studies open up the possibility that eCBs may have opposite and complementary regulatory effects, namely, while their signaling lead to transient or LTD via presynaptic CB_1_ receptors in local homoneuronal synapses (Wilson and Nicoll, 2001; Chevaleyre and Castillo, 2003), they transiently (Navarrete and Araque, 2010) or persistently (Gómez-Gonzalo et al., 2015) potentiate synaptic transmission at more distant synapses through activation of astrocytes. It is unclear whether these opposite mechanisms might coexist *in vivo* or whether they occur only in specific physiological or pathophysiological circumstances.

While a lot has been learned about the role of astrocytes on eCB-mediated synaptic plasticity, how exactly gliotransmitters are released from astrocytes is uncertain but it could involve vesicular exocytosis in some cases. Using the light chain of tetanus toxin and Evans blue, an inhibitor of the vesicular glutamate transporter, Min and Nevian (2012) demonstrated that eCB-mediated t-LTD requires the SNARE-dependent exocytosis of astroglial glutamate. Likewise, other study showed that eCBs trigger release of ATP and D-serine from neocortical astrocytes by SNARE-complex-dependent mechanism, with ATP being crucial for LTP facilitation (Rasooli-Nejad et al., 2014). Although it is well-accepted that [Ca^2+^]_i_-dependent vesicular fusion of either large or small synaptic-like vesicles (Araque et al., 2000; Bezzi et al., 2004; Martineau et al., 2008, 2013; Kang et al., 2013) lead to glutamate and D-serine release from astrocytes, the involvement of alternative non-vesicular pathways in eCB-mediated plasticity deserve more investigation. One alternative mechanism may reside in the opening of hemichannels, either directly as a route for diffusion or indirectly by favoring Ca^2+^ entry that subsequently activates other [Ca^2+^]_i_-dependent gliotransmitter release pathways (Montero and Orellana, 2015). Certainly, hemichannels formed by Cx43, the predominant channels of their kind in astrocytes, have already been associated with the release of diverse gliotransmitters such as glutamate (Ye et al., 2003; Orellana et al., 2011b), ATP (Stout et al., 2002; Kang et al., 2008; Chever et al., 2014), D-serine (Meunier et al., 2017), glutathione (Rana and Dringen, 2007) and lactate (Karagiannis et al., 2016). A while ago, it was showed that lowering extracellular Ca^2+^ to concentrations that take place during neuronal bursting activity, causes ATP efflux via astroglial Cx43 hemichannels, which subsequently strengths inhibitory transmission by activation of neuronal P2Y_1_R (Torres et al., 2012). The latter study brought to light the idea that gliotransmitter release linked to the physiological opening of astroglial hemichannels may regulate synaptic transmission. This was demonstrated later on by Chever et al. (2014), who found that constitutive function of astroglial Cx43 hemichannels contributes to ATP release and tuning of basal excitatory synaptic transmission in the hippocampus.

Additionally, when studied in basal conditions, the opening of astroglial Cx43 hemichannels enhances the amplitude of slow oscillations in mitral cells of the olfactory bulb (Roux et al., 2015) and is essential for fear memory consolidation in the basolateral amygdala (BLA), a brain region crucial for anxiety and emotional memory processing (Stehberg et al., 2012). The last-mentioned study revealed that microinjection of the BLA with TAT-L2, a peptide that specifically blocks Cx43 hemichannels, does not affect short-term memory, but fully induces amnesia towards an auditory fear conditioning paradigm (Stehberg et al., 2012). TAT-L2, along with Gap19, are the most predominant pharmacological tools used to inhibit hemichannel function without affecting gap junctional communication (Iyyathurai et al., 2013). TAT-L2 is a cell-permeable mimetic peptide of the so-called L2 cytoplasmic loop region of Cx43, whereas Gap19 is a smaller nonapeptide derived from it (Iyyathurai et al., 2013). Remarkably, the TAT-L2-mediated amnesic response was prevented by co-infusing a mixture of gliotransmitters together with the peptide, including glutamate, D-serine, glycine, lactate, ATP and glutamine. Likewise, Cx43 hemichannels appear to be critical for spatial memory. Using the spontaneous alternation Y maze training, a recent study reported that blockade of astroglial Cx43 hemichannels impairs hippocampal-dependent short-term spatial memory, but not working memory (Walrave et al., 2016). Decisive evidence establishing the involvement of astroglial hemichannels in regulating synaptic transmission came from a recent study of Giaume’s group. They observed that Cx43 hemichannel inhibition and clamping [Ca^2+^]_i_ in astrocytes decreases NMDA but not AMPA postsynaptic currents in the prefrontal cortex (Meunier et al., 2017). Furthermore, electrophysiological experiments of high frequency stimulation revealed D-serine release linked to astroglial Cx43 hemichannel opening is crucial for LTP of NMDA or AMPA synaptic currents (Meunier et al., 2017). This study uncover a possible mechanism to explain previous findings showing that exogenous administration of D-serine revert LTP impairment caused by inhibition of [Ca^2+^]_i_ oscillations in astrocytes (Henneberger et al., 2010).

**Figure 3:**
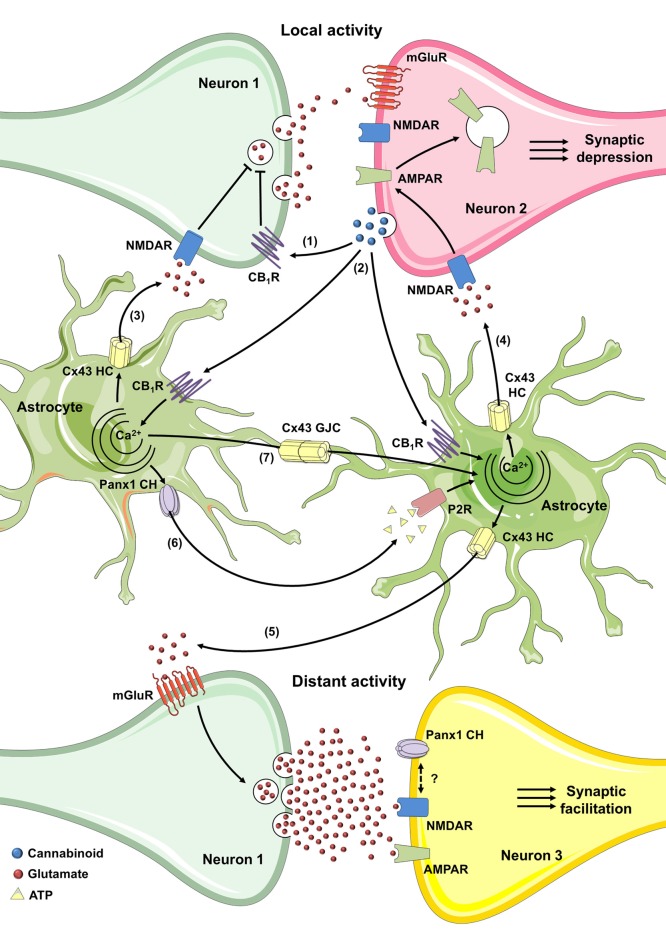
Possible roles of connexin- and pannexin-based channels in CB-mediated synaptic plasticity through activation of astrocytes. In the hippocampus, the activity-dependent production of eCB triggers a decrease of neurotransmitter release through the stimulation of presynaptic CB_1_Rs in homoneuronal synapses (1). In the cortex and hippocampus, the coincidence of postsynaptic metabotropic glutamate receptor (mGluR) activation during synaptic activity and Ca^2+^ influx (not depicted) caused by postsynaptic back-propagating action potentials evoke the release of eCBs (2). The latter stimulates astroglial CB_1_ receptors and could elicit the Ca^2+^-dependent release of glutamate or D-serine through Cx43 hemichannels (3), leading to the activation of presynaptic NMDA receptors (NMDARs) and subsequent induction of spike timing dependent long-term depression (t-LTD; Min and Nevian, 2012; Andrade-Talavera et al., 2016). Alternatively, in the hippocampus, astroglial CB_1_ receptor activation and rise of cytoplasmic Ca^2+^ may cause the Cx43 hemichannel-dependent release of glutamate (4) which, through the stimulation of postsynaptic NMDAR, elicits the internalization of AMPARs and further t-LTD (Han et al., 2012). At the other end, the eCB-mediated increase in cytoplasmic Ca^2+^ may trigger the release of glutamate through Cx43 hemichannels (5) that, acting on mGluRs induces lateral potentiation of synaptic transmission at distant synapses (Navarrete and Araque, 2010; Gómez-Gonzalo et al., 2015). The interacting coupling between NMDARs and Pannexin 1 (Panx1) channels could be a possible mechanism to potentiate the above response. Finally, purinergic signaling mediated by ATP released via Panx1 channels (6) along with gap junctional communication (7) among astrocytes could favor the widespread of eCB-mediated intracellular Ca^2+^ responses.

Overall, the above studies provide a solid picture about the role of astroglial hemichannels in synaptic transmission and thus their involvement on eCB-mediated neural plasticity could be critical. How hemichannels may contribute to the eCB-dependent synaptic dialog between astrocytes and neurons? Given that postsynaptic eCBs cause the [Ca^2+^]_i_-dependent release of glutamate and D-serine from astrocytes (Navarrete and Araque, 2008, 2010; Andrade-Talavera et al., 2016), one may question whether Cx43 hemichannels are involved in this process. In this line, a recent work has shown that eCBs may activate astrocytic hemichannels under basal conditions. Using two-photon *in vivo* microscopy, Vázquez et al. (2015) detected an increased basal activity of astroglial Cx43 hemichannels in the cortex of FAAH-null mice, which possess 15-fold augmented endogenous brain levels of AEA (Cravatt et al., 2001). Equivalent results were observed when AEA was directly applied in the mouse cortex (Vázquez et al., 2015), attributing to eCBs the ability of stimulate basal activity of astroglial hemichannels in the normal brain. It is noteworthy that CB_1_-dependent elevation of [Ca^2+^]_i_ in astrocytes relies on PLC activation and further IP_3_ production (Navarrete and Araque, 2008), the latter being a well-recognized pathway involved in the activation of Cx43 hemichannels (De Bock et al., 2012; Orellana et al., 2012a; Bol et al., 2017), including in astrocytes (Alvarez et al., 2016). Indeed, the opening of Cx43 hemichannels respond to changes in cytoplasmic Ca^2+^ according to a bell-shaped “convex-up” pattern, with maximal activity in the 500 nM range and decreasing activities at both higher and lower [Ca^2+^]_i_ (De Bock et al., 2012). Dye uptake and single-channel recordings have been used to prove this feature in astrocytes, glioma cells and other cells types (De Vuyst et al., 2009; De Bock et al., 2012; Wang et al., 2013b; Bol et al., 2017; Meunier et al., 2017). This evidence places the hemichannels as possible candidates to mediate directly or indirectly the eCB-dependent release of glutamate, D-serine or ATP from astrocytes, the latter being potentially significant for neuronal synaptic function (Figure 3).

Another angle not mentioned so far is the eventual contribution of connexin/pannexin-dependent widespread of astrocyte Ca^2+^ responses in gliotransmission and subsequent CB-mediated potentiation of heterosynaptic plasticity at distant synapses (Navarrete and Araque, 2010; Gómez-Gonzalo et al., 2015). Although ATP seems released through different pathways (Zhang et al., 2007; Kreft et al., 2009), several studies suggests that hemichannels and pannexons are the major contributors to this phenomenon in astrocytes (Stout et al., 2002; Kang et al., 2008; Suadicani et al., 2012). Noteworthy, Panx1 channel-dependent release of ATP relies on protein-protein interactions between P2X_7_Rs and Panx1 (Locovei et al., 2007). In fact, Panx1 co-immunoprecipitates with P2X_7_Rs (Pelegrin and Surprenant, 2006; Silverman et al., 2009), and proline 451 in their C-terminal tails has been involved in this interaction (Iglesias et al., 2008; Sorge et al., 2012). P2YR stimulation also may mediate ATP efflux from astrocytes by activating the PLC/IP_3_/Ca^2+^-dependent opening of hemichannels and pannexons, as has been previously demonstrated for other cell types (Locovei et al., 2006; Orellana et al., 2012a). In addition, because hemichannels are permeable to Ca^2+^ (Sánchez et al., 2009; Schalper et al., 2010; Fiori et al., 2012), their dependency on [Ca^2+^]_i_ may contribute to perpetuate [Ca^2+^]_i_-induced Ca^2+^ entry pathways associated to ATP release. Altogether, these data highlight that ATP signaling via purinergic receptors, hemichannels and pannexons may represent a plausible mechanism for underlying the CB_1_-dependent astroglial modulation of synaptic transmission and plasticity (Figure 3). In agreement with this notion, ATP released from eCB-stimulated astrocytes directly activates post-synaptic P2XRs, facilitating LTP due to downregulation of synaptic and extra-synaptic GABA receptors in cortical pyramidal neurons (Rasooli-Nejad et al., 2014). Desensitization of purinergic P2XRs/P2YRs and degradation of extracellular ATP by exonucleases may turn off in part ATP-dependent widespread of Ca^2+^ responses in astrocytes (Fields and Burnstock, 2006). Other negative feedback loops may reside in the direct counteracting action of ATP on Panx1 channels (Qiu and Dahl, 2009), as well as the inhibition of Cx43 hemichannels by [Ca^2+^]_i_ over 500 nM (Meunier et al., 2017). Future studies will uncover whether opening of hemichannels and pannexons may contribute to astrocyte signaling during eCB-mediated synaptic transmission and plasticity.

In addition to contributing to astroglial-mediated release of gliotransmitters, Panx1 channels may also regulate synaptic communication given its broad expression and functionality in neurons (Thompson, 2015). Precisely, Panx1 is mainly found in the postsynaptic density of excitatory neurons (Zoidl et al., 2007) and couple NMDARs (Weilinger et al., 2012), making it well positioned to participate in eCB-mediated synaptic plasticity. Two independent groups have showed that acute hippocampal slices from adult Panx1 KO mice exhibit a significant increase in synaptic transmission, as measured in input-output curves at the hippocampal Schaffer-collateral CA1 synapse (Prochnow et al., 2012; Ardiles et al., 2014). As either adenosine application or blockade of NMDARs restored normal synaptic transmission (Prochnow et al., 2012), it is possible that loss of Panx1 may trigger extracellular adenosine depletion, thus favoring activation of postsynaptic NMDARs. Supporting this idea, stimulation of adenosine A1 receptors blunt the release of glutamate from pre-synaptic terminals (Dunwiddie and Masino, 2001) and hyperpolarizes post-synaptic neurons via ATP-sensitive K^+^ channels (Kawamura et al., 2010). Up to now, it is unclear whether a similar mechanism might account for transient or long-term eCB-mediated synaptic depression in the hippocampus or cortex (Han et al., 2012; Min and Nevian, 2012; Andrade-Talavera et al., 2016).

## Concluding Remarks and Future Directions

As we noted before, it seems that the outcome of CBs in the functional activity of hemichannels will depend on the physiological status of the brain. Namely, under physiological conditions, eCBs may induce the controlled opening of astrocyte hemichannels and the consequent release of gliotransmitters. This may be particularly relevant for transient or long-lasting mechanisms of synaptic transmission and plasticity evoked by eCBs. At the other end, in the inflamed brain, eCBs may counteract the dysfunctional opening of hemichannels by triggering a large-scale of anti-inflammatory pathways that result in the inhibition of hemichannels and stimulation of astroglial coupling. The exact scenario and cellular cascades by which eCBs lead to this dual opposite regulation of hemichannels remain uncertain. Nevertheless, part of the explanation may rely in that eCBs may have different outcomes depending on which CB receptor they are exerting their actions. Additionally, the altered expression and production of CB receptors and eCBs during pathological conditions, respectively, may trigger different pathways that oppositely regulate the function of hemichannels. Considering the recent evidence linking the activity of GJCs, hemichannels and pannexons with major depression, addiction, autism, epilepsy and schizophrenia (Sarrouilhe et al., 2017), one may question whether the dialog between connexin/pannexin-based channels and the CB system may also impact the pathogenesis and progression of psychiatric disorders. At the other end, given the well-established role of eCB receptors in adult neurogenesis (Prenderville et al., 2015) and because connexin/pannexin function has been implicated in this process (Rozental et al., 1998; Swayne et al., 2010; Liebmann et al., 2013; Salmina et al., 2014; Swayne and Bennett, 2016), it would be interesting to unveil how the interaction between both pathways may impact neural progenitor proliferation, neuronal differentiation, maturation and survival. The mechanisms involved in all these events need to be studied further to better understand their biological implications for developing novel therapies against different brain diseases.

## Author Contributions

JAO: conceived and designed the major ideas developed in the manuscript. VCL, CAS, RG-G, EFD, GIG and JAO: reviewed the literature and designed the figures; wrote and edited the manuscript. All authors read and approved the final manuscript.

## Conflict of Interest Statement

The authors declare that the research was conducted in the absence of any commercial or financial relationships that could be construed as a potential conflict of interest.
